# Triple Burden of HIV, HBV and HDV in Adults with Childhood Parenterally Acquired Infections: A Romanian Single-Center Study

**DOI:** 10.3390/pathogens14121261

**Published:** 2025-12-10

**Authors:** Manuela Arbune, Alina-Viorica Iancu, Monica-Daniela Padurariu-Covit, Alina Plesea-Condratovici, Anca-Adriana Arbune, Catalin Plesea-Condratovici

**Affiliations:** 1 Clinical Medical Department, Medicine and Pharmacy Faculty, “Dunarea de Jos” University, 800008 Galati, Romania; manuela.arbune@ugal.ro; 2 Infectious Diseases Clinic Hospital, 800179 Galati, Romania; alina.iancu@ugal.ro; 3 Morphological and Functional Sciences Department, Medicine and Pharmacy Faculty, “Dunarea de Jos” University, 800008 Galati, Romania; catalin.plesea@ugal.ro; 4 Doctoral School of Biomedical Sciences, “Dunarea de Jos” University, 800008 Galati, Romania; monica.padurariu@ugal.ro; 5 Medical Department, Medicine and Pharmacy Faculty, “Dunarea de Jos” University, 800008 Galati, Romania; alina.plesea@ugal.ro; 6 Neurology Department, Fundeni Clinical Institute, 022328 Bucharest, Romania; 7 Multidisciplinary Integrated Center of Dermatological Interface Research (MIC-DIR), “Dunărea de Jos” University, 800008 Galati, Romania

**Keywords:** HIV, HBV, HDV, co-infections, Romanian HIV cohort, childhood infection, tenofovir

## Abstract

Background: Co-infections with HIV, HBV, and HDV pose significant public health challenges, especially in populations exposed parenterally. Romania hosts a unique pediatric HIV cohort of individuals born 1987–1995 who acquired HIV iatrogenically. This study assessed the prevalence, hepatic impact, and management of HIV–HBV–HDV co-infection in 130 long-term survivors from Galați County. Methods: Patients underwent clinical, laboratory, and FibroScan assessments. HBV and HDV serology and viral loads were measured, and antiretroviral therapy regimens, including tenofovir-based therapies, were reviewed. Entecavir or Bulevirtide was applied when indicated. Results: HBV infection was present in 57.7% of cohort patients versus 20% in non-cohort PLWH, and HDV co-infection in 7.7% of cohort patients. Hepatic fibrosis increased from HBV-uninfected to HBV/HDV co-infected individuals. HIV impairs viral clearance and exacerbates liver injury via immune dysregulation and chronic inflammation. Despite TDF-based ART, replicative HBV was detected in eight patients, managed with Entecavir. Bulevirtide therapy for HDV was initiated in eligible patients, with minor adverse events. Conclusions: Pediatric HIV cohort survivors show high rates of HBV and HDV co-infection and progressive hepatic fibrosis. Optimized antiviral therapy and adherence support are essential to control viral replication and reduce liver-related complications.

## 1. Introduction

Viral infections with the human immunodeficiency virus (HIV), hepatitis B virus (HBV), and hepatitis D virus (HDV) constitute major global public health challenges, owing to the high prevalence of coinfections and their synergistic progression. The coexistence of these infections results from their common transmission routes—parenteral, sexual, and perinatal—especially within vulnerable groups.

Worldwide, approximately 38 (40.8) million people are living with HIV, with about 1.5 (1.3) million new cases diagnosed annually, and an estimated 63,000 deaths attributed to acquired immunodeficiency syndrome (AIDS)-related diseases [1].

In the regional context of Eastern Europe, the incidence of HIV infection was reported at 5.3 per 100,000 population in 2023. Romania is considered a low-prevalence country (among individuals aged 15–49 years), with an estimated prevalence of 0.10% [2,3]. According to the most recent national HIV/AIDS surveillance report, Romania registered 18,768 people living with HIV as of 31 December 2024, out of a cumulative total of 28,793 diagnosed cases since 1985. Current epidemiological trends (2007–2024) show that heterosexual transmission represents the predominant route, followed by male-to-male sexual contact (MSM) and parenteral transmission among people who inject drugs, while perinatal transmission accounts for a much smaller proportion of new diagnoses [4].

Romania has a unique epidemiological profile, shaped by a pediatric HIV cohort consisting of children born between 1987 and 1995 who acquired health care associated infections, primarily through unsafe injections and contaminated blood transfusions, mostly during their first year of life. Of the more than 10,000 children infected during the 1990s, a considerable number have survived into adulthood, over 5000 long-term survivors remain alive today, now reaching adulthood and presenting various comorbidities and coinfections, forming a distinctive population with specific healthcare needs [4,5,6,7].

Recent global estimates indicate that between 5% and 20% of people living with HIV (PLWH) have concurrent chronic HBV infection, while HDV superinfection affects approximately 7% of those with chronic HBV. However, the frequency of these co-infections varies according to regional prevalence data [8].

In Europe, the highest rates of HDV endemicity are observed in the Eastern and Mediterranean regions; however, migration from Eastern countries to Central and Western Europe is altering the epidemiology of this infection. According to European surveillance reports, HDV is reported predominantly among individuals co-infected with HIV/HBV, intravenous drug users, migrants, and patients with severe HBV infection [9].

In Romania, the prevalence of HBV infection is estimated at 3–5% of the population, accounting for approximately 600,000–700,000 chronic carriers [9,10,11]. Furthermore, approximately 10,000–15,000 patients are affected by HDV [11,12]. The introduction of HBV vaccination in 1995 significantly reduced the incidence of new cases of hepatitis B and subsequently coinfections with HDV [13]. However, many patients in the pediatric HIV cohort could remain co-infected with HBV or double HBV and HDV.

The aim of this study is to evaluate the prevalence, hepatic impact, and management options of HIV/HBV/HDV co-infection among survivors from the pediatric cohort in Galați County. The specific objectives are to determine the prevalence of HBV and HBV/HDV co-infections in this cohort compared with non-cohort HIV patients from the Galați region, and to identify factors associated with different HBV co-infection patterns within the pediatric HIV cohort. The study was conducted in accordance with the Declaration of Helsinki and approved by the Ethics Committee of Infectious Diseases Clinic Hospital “Sf. Cuv. Parascheva” Galaţi (protocol code 21/18.09.2025).

## 2. Materials and Methods

### 2.1. Study Design

This study retrospectively assessed the prevalence of HBV and HBV/HDV co-infections and their impact on liver function in patients from the paediatric HIV cohort monitored in Galați County. The study was conducted at the “Sf. Cuv. Parascheva” Clinical Hospital of Infectious Diseases in Galați, within the Day Clinic for HIV/AIDS patients, during the first semester of 2025.

### 2.2. Patients’ Selection

Eligible cases were born between 1987 and 1995, diagnosed before age 14, and had documented parenteral exposures in early life (blood transfusions, hospitalizations, parenteral treatments, or other invasive procedures). Infections acquired perinatally, through injection drug use, or via sexual transmission were excluded. Maternal HIV testing was systematically performed to rule out perinatal transmission. Individuals who first tested HIV-positive after age 14 and were sexually active were classified as having infections of uncertain transmission route and were excluded from the analysis.

### 2.3. Data Collection

In accordance with local protocols for monitoring PLWH, all patients were screened at the time of HIV diagnosis for hepatitis C virus antibodies (HCV-Ab) and hepatitis B virus markers (HBsAg and HBcAb).

Surviving patients who presented during the first semester of 2025 underwent routine annual HIV follow-up, including a comprehensive clinical examination, FibroScan assessment, and laboratory testing consisting of CD4 cell count, HIV-RNA, platelet count, ALT, AST, AFP, lipid profile. We revised HBV serology (HBsAg, HBcAb, HBsAb) and HDV antibodies (HDV-Ab), if HBsAg-positive. HBsAb levels were also assessed, with titers > 10 IU/mL considered protective. Patients positive for HBV markers (HBsAg and/or HBcAb) have been virological assessed HBV-DNA, and additional HDV RNA in HDV-Ab-positive individuals.

Current antiretroviral therapy (ART) regimens were analysed, with particular attention to those tenofovir- containing therapies (tenofovir disoproxil fumarate or alafenamide; TDF/TAF). Additional data extracted from clinical records included year of HIV diagnosis, disease stage (AIDS vs. non-AIDS according to the 1994 CDC clinical–immunological classification), and treatment history.

### 2.4. Laboratory Testing

HBV serological markers were determined using the ELISA method with the STAT FAX 2100 microplate reader (Awareness Technology Inc., Palm City, FL, USA). Viral loads of HBV DNA, HDV RNA, and HIV RNA were quantified using the GeneXpert system (Cepheid, Sunnyvale, CA, USA) operated with GeneXpert Dx System Software version 6.5.

### 2.5. Assessment of Liver Status

Hepatic fibrosis was evaluated using vibration-controlled transient elastography (VCTE) and estimated through APRI and FIB-4 scores. Liver stiffness was measured with the FibroScan 630 Expert device (Echosens, Paris, France) following standard examination procedures. The median of 10 valid measurements was considered for analysis. Liver stiffness was interpreted using the following cut-offs: <6.6 kPa or F0–F1 (no or mild fibrosis); 6.6–7.9 kPa or F2 (moderate fibrosis); 8–9.9 kPa or F3 (severe fibrosis); ≥10 kPa or F4 (cirrhosis).

The APRI score was calculated as follows: APRI = [(AST/ULN) × 100]/platelet count (10^9^/L), where ULN is the upper limit of normal. An APRI score > 1.0 predicts cirrhosis with 76% sensitivity and 72% specificity, while an APRI score > 0.7 predicts significant fibrosis with 77% sensitivity and 72% specificity [14,15].

The FIB-4 score was calculated as follows: FIB-4 score = {[age (yr) × AST (U/L)]/[platelet count (10^9^/L) × ALT (U/L)]}. FIB-4 values < 1.45 have a negative predictive value of 90% for advanced fibrosis, whereas values > 3.25 indicate advanced fibrosis with a specificity of 97% and a positive predictive value of 65% [16,17,18].

### 2.6. Statistical Analysis

Collected data were analyzed statistically using XLStat software (Addinsoft, Paris, France), version 2022.4.5. Descriptive statistics were used to summarize demographic and clinical characteristics. Categorical variables, including serological profiles and co-infection status, were expressed as absolute numbers and percentages. Continuous variables were reported as mean ± standard deviation (SD) or median (interquartile range, IQR), depending on distribution. Comparisons between groups were performed using chi-square or Fisher’s exact test for categorical variables and *t*-test or Mann–Whitney U test for continuous variables, depending on data distribution. For comparisons involving more than two groups with small expected frequencies, the Fisher-Freeman-Halton exact test was applied. All analyses were two-tailed, and *p*-values < 0.05 were considered statistically significant. Where appropriate, 95% confidence intervals (CI) were calculated to estimate the precision of proportions and means.

## 3. Results

### 3.1. HIV Transmission Patterns and Definition of the Paediatric Cohort

As of 31 January 2025, a total of 395 people living with HIV (PLWH), aged 19–77 years, were under follow-up at the Day Clinic for HIV/AIDS monitoring and treatment. Among these, 175 individuals were aged 30–38 years, corresponding to birth years 1987–1995, which aligns with the Romanian paediatric HIV cohort.

**Figure 1:**
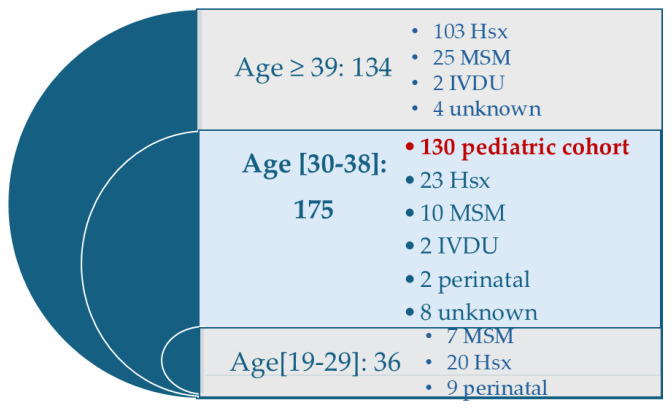
Distribution of PLWH attending the Galati Day Clinic according to age group and HIV transmission patterns. Legend: Hsx: Heterosexually; MSM: men who have sex with men; IDVU: intravenous drug users.

Epidemiological data analysis identified 130 PLWH from the paediatric cohort (N = 130), representing 74.3% of patients within this age group and 33% of all cases registered at the clinic [Figure 1]. Percentages reported in the subsequent analyses are calculated based on this cohort size. For comparison, the non-cohort sample (Ns) comprised 245 PLWH aged 19–77 years, outside the pediatric cohort.

The figure shows the number of individuals in each age category and the corresponding HIV transmission patterns. The pediatric cohort (*n* = 130) is highlighted.

### 3.2. Prevalence of HBV and HBV/HDV Co-Infections

The prevalence of HBV infection among pediatric cohort patients was 57.7%, significantly higher than the 20% observed in non-cohort PLWH, as indicated by HBcAb and HBsAg serological markers. HBV/HDV co-infection (HDV-Ab) was present in 7.7% of the cohort, while HDV markers were absent in all non-cohort patients. The high prevalence of HBV and HDV infections in the cohort reflects increased exposure and a unique epidemiological context for parenterally transmitted co-infections during the likely period of HIV acquisition (1987–1992) [Table 1].

All cohort and non-cohort PLWH tested negative for HCV antibodies (HCV-Ab).

### 3.3. Current Characterization of HIV Pediatric Cohort

We identified 130 PLWH originating from the pediatric cohort, with a current mean age of 36.13 ± 0.99 years. The mean duration since HIV diagnosis was 27.52 ± 4.15 years, and 78.5% were classified as having AIDS. The sex distribution was balanced (female-to-male ratio 1:1), and the rural-to-urban residence ratio was 1.06. Regarding educational level, 24% had completed primary education, 44% secondary education, and 32% high school or higher. Reported risk behaviors included smoking (52%) and alcohol consumption (19%). Obesity (body mass index > 30 kg/m^2^) was documented in 10% of participants. At the time of the study, no women with an ongoing pregnancy were identified.

All patients were receiving antiretroviral therapy, with 82% achieving complete viral suppression and a mean CD4 count of 581 ± 313/mm^3^. Among current therapeutic regimens, 81% included an agent with antiviral activity against HBV: 37% Tenofovir disoproxil fumarate (TDF) and 44% Tenofovir alafenamide (TAF). It should be noted that patients in the cohort had been exposed to multiple antiretroviral therapeutic regimens, with a median of five regimens [3,12].

The most frequent laboratory abnormalities among paediatric cohort PLWH were dyslipidaemia (37%), cholestasis (26%), cytolysis (17%), elevated C-reactive protein (15%), and thrombocytopenia (10%). No patients exhibited clinically significant elevations in alpha-fetoprotein (AFP) [Table A1].

Hepatic elastography showed fibrosis distribution as follows: F0–F1, 81%; F2–F3, 14%; and F4, 5%. These results were concordant with fibrosis scores derived from APRI (0.480 ± 0.534; range 0.153–4.527) and FIB-4 (1.00 ± 0.88; range 0.29–9.89) [Table 2].

Univariate analysis demonstrated that serologic HBV co-infection patterns significantly impacted fibrosis as assessed by FibroScan and FIB-4 scores, but not by the APRI score, with fibrosis progressively increasing from mono-infected PLWH to those with occult HBV co-infection, active HBsAg co-infection, and finally HBV and HDV co-infection. A separate comparison between HBV mono-infection (HBsAg+/Anti-HDV−) and HBV/HDV co-infection confirmed that differences were largely not statistically significant, except for urban living and institutionalization (abandon), highlighting higher HDV risk among individuals from children’s care institutions. No consistent correlations were observed with biochemical liver markers [Table A2].

Smoking may represent an additional risk factor associated with institutionalization, potentially influencing fibrosis progression. Furthermore, current antiretroviral therapy regimens were tailored according to the patient’s serologic profile.

### 3.4. Virological Assessment of HBV and HDV

Within 130 evaluated patients from HIV paediatric cohort, we found diverse HBV serological profiles. A total of 42.3% (55/130) of patients were uninfected, with HBsAg–/HBcAb—serological profiles. HBsAb determination showed values > 10 IU/mL in 9.3% (12/130) of PLWH, indicating either post-vaccination immune control (only 9% of uninfected patients) or seroconversion following natural infection (9.3% of patients with HBV infection markers). Vaccination history against HBV was not available for all PLWH. Nevertheless, 35% of patients are eligible for HBV vaccination based on demonstrated susceptibility.

The prevalence of HBV infection, defined by the presence of HBcAb ± HBsAg, was 57.7% (75/130). The HBsAg−/HBcAb+ profile was observed in 34.5% (45/130) of PLWH, indicating occult infection following HBsAg seroconversion. Persistent HBV infection, whether active or inactive, was represented by the HBsAg+/HBcAb+ profile, present in 23.1% (30/130) of patients.

**Figure 2:**
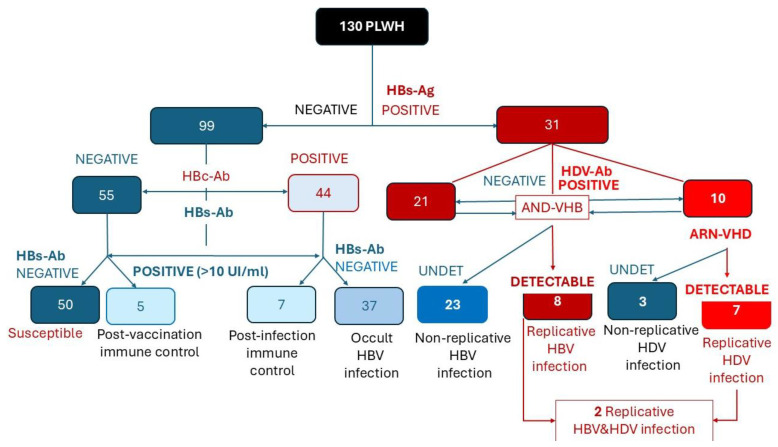
Flow chart illustrating the HBV and HDV serological and virological evaluation among 130 PLWH from the pediatric cohort. Red shades indicate B ± D co-infections, while blue shades represent individuals without current hepatitis virus co-infections.

HDV co-infection was identified in 10 patients, representing 7.7% of the total HIV cohort and 33.3% of those with persistent HBsAg [Figure 2].

Viral replication was assessed in all patients with HDV antibodies and in those with markers of occult or active HBV infection. Among HBsAg-positive PLWH, 25.8% exhibited viral replication HBV-DNA. HDV- RNA was detectable in 70% of HDV infections (7/10 co-infected patients) [Figure 2].

The diagram summarizes the classification of patients based on HBsAg, HBc-Ab, HBs-Ab, HDV-Ab, HBV-DNA, and HDV-RNA results, distinguishing susceptible, immune, occult, non-replicative, and replicative HBV/HDV infection stages.

Although most co-infected patients received tenofovir-containing antiretroviral regimens, HBV replication occurred in a subset of patients with detectable HIV viremia, possibly reflecting suboptimal adherence not directly assessed.

## 4. Discussion

### 4.1. Epidemiology

The characteristics of HIV/HBV/HDV coinfection in the pediatric HIV cohort reflect the early 1990s dramatic epidemiological context in Romania, when social, political, and economic factors contributed to the parenteral transmission of HIV, HBV, and HDV among young children. In 1990, when the political regime changed and diagnostic testing became available, more than 1000 children were diagnosed in Romania, 97% of whom were under the age of 3 years, and 62% were institutionalized. A proportion of the children infected during that period have since evolved into a population of long-term survivors [19,20,21].

HIV/HBV/HDV coinfections are less well documented in Romania, as access to HDV testing has been limited in the past, and current epidemiological reporting systems have not required such information. A few studies conducted more than 15 years ago reported variable rates of HIV/HBV/HDV coinfection across different regions of the country: 5.6% in Bucharest, 9.37% in Craiova, and 23.8% in Galați [22,23,24].

Compared with the data reported in our region (Galați) more than 10 years ago, the frequency of HDV infection has decreased from 23.8% to 7.7%. This decrease may be explained by attrition of pediatric cohort patients due to premature death or migration. In the previous study, a retrospective analysis over a 10-year period (2000–2012) indicated a mortality rate of 11%, with chronic liver disease associated with HBV/HDV coinfection potentially contributing to these fatalities [23]. Lamivudine use in most first-line regimens for children in our cohort, as previously reported, carried a risk of HBV resistance, with implications for future therapy and the potential transmission of resistant strains [25]. The introduction of tenofovir in Romania after 2020 has improved HBV control and, indirectly, HDV control compared with earlier periods [23,26].

It should be noted that HDV infection has remained limited to patients from the cohort, particularly among those who were abandoned in childhood and institutionalized, while individuals outside the cohort have not been specifically affected.

### 4.2. Long-Term Outcomes

Early-life acquisition of HBV infection, evidenced by persistent HBsAg in children infected or exposed to HIV during the first years of life, combined with immunologic immaturity, likely fosters immune tolerance and promotes chronic HBV infection, also favoring HDV persistence in co-infected individuals [25,27,28].

Most evidence indicates that chronic HBV does not affect HIV progression or suppression [25].

HIV impacts HBV and HBV/HDV infections through impaired CD8+ T cell activation necessary for clearing infected hepatocytes, diminished IFN-γ production with antiviral effects, and altered B cell-mediated humoral responses, such as anti-HBs antibody production [29]. Consequently, immunosuppression decreases viral clearance and increases viral replication. While HIV may theoretically attenuate immune-mediated liver injury, chronic inflammation and immune dysregulation continue to exacerbate hepatic damage. Antiretroviral therapy partially restores immune function, it does not fully reverse these effects, maintaining ongoing liver disease risk [29,30].

HIV/HBV/HDV coinfection severely impacts the liver by impairing viral clearance, prolonging hepatitis, accelerating fibrosis, and increasing hepatic morbidity and mortality, as reflected in acute hepatitis flares and rapid progression to cirrhosis and decompensation [28,29,30]. The effect of HIV/HBV/HDV coinfection hepatocellular carcinoma (HCC) remains unclear, with some studies indicating increased risk and others showing no significant association [26,28,31]. Notably, EASL guidelines identify HIV coinfection as a risk factor for fibrosis progression [31].

The ICONA cohort provides valuable insights into HIV-HBV-HDV coinfection, reporting that 18.8% of patients with HIV-HBV coinfection also had HDV superinfection, 67.7% of whom presented replicative forms. HDV superinfection carries a fivefold higher risk of progression to hepatic fibrosis and related complications [32]. In our study, hepatic fibrosis was more pronounced in HDV-positive patients, although the difference did not reach statistical significance, likely due to the small sample size (small-sample bias).

### 4.3. HIV/HBV/HDV Management

According to national procedures and current guideline recommendations, several targeted local interventions were identified [31,33,34].

First intervention must be HBV vaccination, as susceptibility was documented in 35% of patients.

All HIV/HBV coinfected patients in our cohort received TDF- or TAF-based ART. Enhancing and maintaining adherence was implemented to prevent occult HBV reactivation and control active HBV infection. Despite TDF-based regimens, replicative HBV was detected in eight PLWH, including two with triple HIV/HBV/HDV infection, for whom Entecavir was considered to reinforce HBV suppression.

Active HDV infection can be treated in Romania with the recently approved antiviral Bulevirtide. Among seven eligible patients, three initiated therapy, two declined treatments due to its injectable route, one patient is undergoing counselling and therapy is temporarily deferred in one case, due to severe encephalopathy and behavioral issues. Therapeutic outcomes will be evaluated at 24 weeks. A single minor local adverse event occurred at initial administration, without requiring treatment discontinuation.

A recent study from France reported a virological response rate of 50%, with Bulevirtide being safe and without drug interactions [35]. According to EACS recommendation, combination of Bulevirtide and PegInterferon could be considered in our patients, but the duration of the antiviral treatment is still unclear [33,34].

Therapeutic progress in HDV remains limited, with interferon offering modest efficacy and nucleoside analogues target only HBV replication [36]. Novel strategies focus on the viral replication cycle, including Bulevirtide, anti-preS1 monoclonal antibodies, farnesyl transferase inhibitors such as Lonafarnib, and agents disrupting HBsAg production [37,38]. As most studies exclude patients with HIV, ethical reassessment and tailored approaches are needed for triple HIV/HBV/HDV infection.

### 4.4. Study Limitations

The small number of HDV co-infected patients may have led to an underestimation or overestimation of the impact of co-infection on liver fibrosis and other clinical parameters. A multivariable analysis was not feasible due to the very small size of several serological subgroups—especially the HDV-positive group—limiting statistical power and increasing the likelihood that observed associations are influenced by underlying factors. The study focuses on survivors of a unique pediatric cohort exposed parenterally in Romania between 1987 and 1995, which limits the generalizability of the findings to other populations or to patients infected more recently. Incomplete HBV vaccination histories may affect the interpretation of immunity and susceptibility to HBV and HDV. The absence of systematic longitudinal liver assessments prevents a definitive evaluation of HDV’s role in fibrosis progression. Inclusion was limited to patients who attended clinic follow-up, introducing potential survivor bias and excluding individuals who died prematurely or emigrated.

### 4.5. Future Research Directions

Future research should focus on large, multicenter cohort studies to enhance sample size, representativeness, and the characterization of long-term liver outcomes in individuals with childhood-acquired HIV/HBV/HDV coinfection. Clinical trials evaluating the safety and efficacy of emerging HDV therapies in HIV-infected populations are also warranted, given their limited representation in current studies. Additionally, molecular and immunologic investigations into virus–host interactions in triple infection may elucidate mechanisms underlying accelerated liver disease and identify novel therapeutic targets. Expanded epidemiological surveillance and systematic HDV screening in HIV/HBV populations, combined with public health strategies to strengthen HBV vaccination, improve treatment adherence, and enhance access to specialized care, would further support early detection and optimal management of this complex infection.

## 5. Conclusions

HIV–HBV co-infection is highly prevalent in the Romanian pediatric HIV cohort, affecting 57.7% of patients, with persistent HBV in 23.1% and ongoing viral replication in 25.8%. HDV co-infection occurs in 7.7% of the cohort, representing 33.3% of persistent HBV cases, with 70% showing active HDV replication. Early-life HBV acquisition and HIV-related immunosuppression may contribute to viral persistence and HDV superinfection, as suggested by the observed association between detectable HIV viremia and ongoing HBV/HDV replication. Early-life HBV acquisition and HIV-related immunosuppression may contribute to persistent HBV infection and HDV superinfection, as suggested by the association between detectable HIV viremia and ongoing HBV/HDV replication. Maintaining adherence to tenofovir-containing ART is essential to control viral replication and prevent liver disease progression, while HBV vaccination remains indicated for susceptible individuals. Active HDV infection may benefit from novel therapies such as Bulevirtide, alone or combined with pegylated interferon, and targeted interventions are urgently needed to reduce hepatic morbidity in long-term pediatric HIV survivors.

## Figures and Tables

**Table 1:** HBV Serological Profiles in Pediatric HIV Cohort Patients Compared with Other PLWH.

	HIV-Cohort (130)		Non-Cohort (265)		OR	CI95%	*p* *
	N	%	Ns	%			
HBsAg−HBcAb−	55	42.3%	213	80%	0.18	0.11; 0.29	<0.001
HBsAg−HBcAb+	45	34.6%	32	12%	3.85	2.27; 6.57	<0.001
HBsAg+HBcAb+	20	15.4%	21	8%	2.11	1.05; 4.23	0.035
HBsAg+HVDAb+	10	7.7%	0	-	37.1	4.6; 300	<0.001

Legend: N: HIV cohort sample; Ns: HIV non-cohort sample; OR: Odds Ratio; * *p*: Fisher’s exact test.

**Table 2:** Comparison Characteristics of PLWH from Pediatric Cohort According to HBV ± HDV Serological Patterns.

			HBsAg−/Anti-HBc−	HBsAg−/Anti-HBc+	HBsAg+/ Anti-HBc+	HBsAg+/Anti-HDV+	*p* *	*p* **
Overall			55	44	21	10		
Sex	Female	65	33	18	8	6	0.363	0.139
	Male	65	22	26	13	4		
Residence	Urban	63	20	24	10	9	0.041	0.011
	Rural	67	35	20	11	1		
Institutionalized (abandon)	Yes	18	4	8	1	5	0.003	0.001
	No	112	51	36	20	5		
Formal education (years)	≤4	31	14	8	6	3	0.807	0.825
	8	57	23	19	11	4		
	≥12	42	18	17	4	3		
Smoking	Yes	68	22	32	10	4	0.899	0.015
	No	62	33	12	11	6		
Alcohol use	Yes	25	46	36	14	9	0.456	0.502
	No	105	9	8	7	1		
AIDS	Yes	102	42	36	17	7	0.857	0.808
	No	28	13	8	4	3		
CD4 (cells/µL)	>350	105	43	36	16	10	0.353	0.450
	<350	25	12	8	5	0		
ARN-HIV (copies/mL)	<40	107	42	40	16	9	0.897	0.368
	>40	23	13	4	5	1		
Obese	Yes	13	4	5	2	2	0.814	0.653
	No	117	51	39	19	8		
Fib-4	>1.45	15	3	4	5	3	0.770	0.026
	<1.45	115	52	40	16	7		
APRI	>1	9	2	3	3	1	0.704	0.376
	<1	121	53	41	18	9		
Fibroscan	0–1	105	47	38	17	5	0.129	0.025
	2–3	18	7	6	3	2		
	4	7	1	2	1	3		
0.300ARV TAF/TDF	Yes	105	45	31	19	10	0.300	0.047
	No	25	10	13	2	0		

Data are presented as n (number of cases) for each infection pattern: HBsAg−/Anti-HBc−, HBsAg−/Anti-HBc+, HBsAg+/Anti-HBc+, HBsAg+/Anti-HDV+. *p* * refers to the comparison between HBV mono-infection (HBsAg+/Anti-HDV−) and HBV/HDV co-infection (HBsAg+/Anti-HDV+), while *p* ** refers to comparisons across all infection pattern groups (5 categories), calculated using Chi-square test, Fisher’s exact test, or Fisher-Freeman-Halton exact test, as appropriate. Statistical significance was considered at *p* < 0.05.

## Data Availability

Data supporting this study are included within the article and supporting materials.

## Abbreviations

The following abbreviations are used in this manuscript:

Ab	Antibody
AFP	Alpha-fetoprotein
AIDS	Acquired Immunodeficiency Syndrome
ALT	Alanine aminotransferase
AST	Aspartate aminotransferase
APRI	Predictive score of Liver Fibrosis
ART	Antiretroviral therapy
ELISA	Enzyme-Linked Immunosorbent Assay
FIB-4	Predictive score of Liver Fibrosis
HBcAb	Hepatitis B core Antibody
HBsAg	Hepatitis B surface Antigen
HBV	Hepatitis B Virus
HDV	Hepatitis D Virus
HIV	Human Immunodeficiency Virus
MSM	Men Who have Sex with Men
OR	Odds Ratio
PLWH	People Living With HIV
SD	Standard Deviation
TAF	Tenofovir alafenamide
TDF	Tenofovir disoproxil fumarate

## Appendix A

**Table A1:** Biochemical Characteristics of Adults from HIV–Paediatric Cohort.

	NRV	Average ± SD	Median	Max; Min	Out-NRV
CD4	>350/μl	581 ± 313	566	1396; 3	19.2% (25) *
CRP	0–5 mg/L	3.579.60	1	91; 0.01	16.9% (22)
Thrombocyte	150–450 * 10^9^/L	222.89 ± 65.80	220	529; 29	10% (13)
ALT	0–34 U/L	38.87 ± 50.24	27	540; 11	16.9% (22) **
AST	0–31 U/L	32.57 ± 28.08	26	309; 14	17.7% (23) **
AP	40–129 U/L	114.88 ± 43.96	108	467; 55	26.2% (34)
GGT	0–55 U/L	51.05 ± 80.91	28	614; 9.3	17.7% (23)
BT	0.1–1.2 mg/dL	0.650 ± 0.28	0.51	1.9; 0.2	3.8% (5)
ALB	3.2–5.3 g/dL	4.06 ± 0.45	4.67	5.5; 2.01	0.8% (1)
INR	0.80–1.20	1.04 ± 0.12	1.02	1.7; 0.8	6.9% (9)
Col-t	100–200 mg/dL	192.50 ± 41.93	190	315; 105	36.9% (48)
HDL	40–70 mg/dL	49.87 ± 12.77	49	98; 7	17.7% (23)
LDL	50–150 mg/dL	119.60 ± 36.20	116	57; 235	18.5% (24)
TG	50–150 mg/dL	117.62 ± 102.76	91	867; 29	17.7% (23)
AFP	<10 ng/mL	1.73 ± 1.70	1.3	9.74; 0.5	0
Cr	0.6–1.2 mg/dL	0.88 ± 0.19	0.84	2.08; 0.53	4.6% (6)

Legend: * > 350/μL; ** > 1.5 NRV; AFP: Alpha-feto-protein; ALT: Alanine-amino-transferase; AP: Alkaline Phosphatase; AST: Aspartate-amino-transferase; BT: total Bilirubine; CRP: C-Reactive Protein; Cr: Creatinine; Col: Cholesterol; HDL: High density cholesterol; LDL: Low density cholesterol; NRV: Normal Reference Values; SD: Standard Deviation; TG: Triglycerides.

**Table A2:** Comparative Analysis of Biochemical Markers and Serologic Patterns of HBV and HVD Co-Infections in an Adult HIV–Paediatric Cohort.

		HBs−HBc−	HBs−HBc+	HBs+ HBc+	HBs+ HDV+	HBs−HBc− vs. HBs−HBc+			HBs−HBc− vs. HBs+HBc+			HBs−HBc− vs. HBs+HDV+		
						OR	95% CI	*p*	OR	95% CI	*p*	OR	95% CI	*p*
CRP	>5	8	5	6	3	0.75	0.22; 2.48	0.641	2.35	0.71; 7.70	0.158	2.51	0.55; 11.39	0.230
	<5	47	39	15	7									
ALT	>1.5NV	1	10	4	3	2.94	0.95; 9.05	0.060	2.35	0.58; 9.50	0.229	4.28	0.91; 20.0	0.064
	<1.5NV	50	34	17	7									
AP	>NV	10	14	7	3	2.1	0.83; 5.29	0.115	2.25	0.73; 6.90	0.156	1.92	0.43; 8.62	0.390
	≤NV	45	30	14	7									
PTL	<NV	5	5	2	1	0.78	0.21; 2.87	0.709	1.05	0.18; 5.89	0.953	1.11	0.11; 10.65	0.927
	>NV	50	39	19	9									
CD4	>200	46	39	17	10	1.52	0.47; 4.90	0.681	0.831	0.22; 3.05	0.781	-	-	0.168
	<200	9	5	4	0									
ARN-HIV	DET	13	4	5	1	3.09	0.96; 9.88	0.056	0.99	0.30; 3.22	0.987	2.78	0.34; 22.32	0.334
	UN	42	40	16	9									

Legend: NV: Normal Value; DET: detectable; UN: Undetectable; CRP: C-Reactive Protein; PTL: Platelets; ALT: Alanin-amino-transpherase; AP: Alkaline Phosphatase.

## References

[1] Joint United Nations Programme on HIV/AIDS (2023). Global HIV & AIDS Statistics—2023 Fact Sheet. Geneva: UNAIDS. https://www.unaids.org/en/resources/fact-sheet?utm_source=chatgpt.com.

[2] WHO Regional Office for Europe, European Centre for Disease Prevention and Control (2024). HIV/AIDS Surveillance in Europe 2024–2023 Data. Copenhagen: WHO Regional Office for Europe. https://www.ecdc.europa.eu/sites/default/files/documents/HIV_Surveillance_Report_2024.pdf.

[3] Institutul Național de Sănătate Publică (INSP), România Analiza Bolilor Transmisibile Aflate în Supraveghere—Raport Pentru Anul 2024. https://insp.gov.ro/download/analiza-bolilor-transmisibile-aflate-in-supraveghere-raport-pentru-anul-2024/.

[4] (2025). Compartimentul Pentru Monitorizarea și Evaluarea Infecției HIV/SIDA în România—INBI “Prof. Dr. M. Balș”. România la 31 Decembrie 2024: Infecția HIV/SIDA—Evoluție și Tendințe. https://www.cnlas.ro/images/doc/2025/ROMANIA%20LA%2031%20DECEMBRIE%20-2024%20-%20site.pdf.

[5] Kozinetz C.A., Matusa R., Cazacu A. (2001). The burden of pediatric HIV/AIDS in Constanta, Romania: A cross-sectional study. BMC Infect. Dis..

[6] Hersh B.S., Popovici F., Jezek Z., Satten G.A., Apetrei R.C., Beldescu N., George J.R., Shapiro C.N., Gayle H.D., Heymann D.L. (1993). Risk factors for HIV infection among abandoned Romanian children. AIDS.

[7] Ruta S., Grecu L., Iacob D., Cernescu C., Sultana C. (2023). HIV-HBV Coinfection—Current Challenges for Virologic Monitoring. Biomedicines.

[8] Dagnaw M., Muche A.A., Geremew B.M., Gezie L.D. (2025). Prevalence and burden of HBV-HIV co-morbidity: A global systematic review and meta-analysis. Front. Public Health.

[9] Demirel A., Uraz S., Deniz Z., Daglilar E., Basar O., Tahan V., Ozaras R. (2024). Epidemiology of hepatitis D virus infection in Europe: Is it vanishing?. J. Viral Hepat..

[10] WHO (2023). Hepatitis B Fact Sheet. Geneva: World Health Organization. https://www.who.int/news-room/fact-sheets/detail/hepatitis-b?utm_source=chatgpt.com.

[11] Iacob S., Gheorghe L., Onica M., Huiban L., Pop C.S., Brisc C., Sirli R., Ester C., Brisc C.M., Diaconu S. (2024). Prospective study of hepatitis B and D epidemiology and risk factors in Romania: A 10-year update. World J. Hepatol..

[12] Grecu L.I., Pavel-Tanasa M., Matei L., Sultana C., Ruta S.M., Grecu R.I., Ursu R.G., Cianga P., Iancu L.S. (2024). Molecular Epidemiology of Hepatitis D Virus in the North-East Region of Romania. Pathogens. Pathogens.

[13] WHO (2024). Global Hepatitis Report 2024: Action for Access in Low- and Middle-Income Countries. Geneva: World Health Organization. https://www.who.int/news-room/fact-sheets/detail/hepatitis-d?utm_source=chatgpt.com.

[14] Lin Z.H., Xin Y.N., Dong Q.J., Wang Q., Jiang X.J., Zhan S.H., Sun Y., Xuan S.Y. (2011). Performance of the aspartate aminotransferase-to-platelet ratio index for the staging of hepatitis C-related fibrosis: An updated meta-analysis. Hepatology.

[15] Chou R., Wasson N. (2013). Blood tests to diagnose fibrosis or cirrhosis in patients with chronic hepatitis C virus infection: A systematic review. Ann. Intern. Med..

[16] Wai C.T., Greenson J.K., Fontana R.J., Kalbfleisch J.D., Marrero J.A., Conjeevaram H.S., Lok A.S.-F. (2003). A simple noninvasive index can predict both significant fibrosis and cirrhosis in patients with chronic hepatitis C. Hepatology.

[17] Vallet-Pichard A., Mallet V., Nalpas B., Verkarre V., Nalpas A., Dhalluin-Venier V., Fontaine H., Pol S. (2007). FIB-4: An inexpensive and accurate marker of fibrosis in HCV infection. comparison with liver biopsy and fibrotest. Hepatology.

[18] Sterling R.K., Lissen E., Clumeck N., Sola R., Correa M.C., Montaner J., Sulkowski M.S., Torriani F.J., Dieterich D.T., Thomas D.L. (2006). Development of a simple noninvasive index to predict significant fibrosis in patients with HIV/HCV coinfection. Hepatology.

[19] Preda M., Manolescu L.C.S. (2022). Romania, a Harbour of HIV-1 Subtype F1: Where Are We after 33 Years of HIV-1 Infection?. Viruses.

[20] Marcu E.A., Dinescu S.N., Pădureanu V., Dumitrescu F., Diaconu R. (2022). Perinatal Exposure to HIV Infection: The Experience of Craiova Regional Centre, Romania. Healthcare.

[21] Arbune M., Calin A.M., Iancu A.V., Dumitru C.N., Arbune A.A. (2022). A Real-Life Action toward the End of HIV Pandemic: Surveillance of Mother-to-Child HIV Transmission in a Center from Southeast Romania. J. Clin. Med..

[22] Ruta S.M., Matusa R.F., Sultana C., Manolescu L., A Kozinetz C., Kline M.W., Cernescu C. (2005). High prevalence of hepatitis B virus markers in romanian adolescents with human immunodeficiency virus infection. J. Int. AIDS Soc..

[23] Arbune M., Georgescu C. (2013). Characteristics of Hepatitis B Co-infection and Disease Evolution in HIV-Positive Paediatric Patients in Romania. Balk. Med J..

[24] Niculescu I., Cupşa A.M., Stoian A.C., Dumitrescu F., Giubelan L.I., Alexandru D.O. (2013). HBV influence on Response to Antiretroviral Therapy in Horizontally HIV-HBV Coinfected Patient during Early Childhood. Curr. Health Sci. J..

[25] Healy S.A., Gupta S., Melvin A.J. (2013). HIV/HBV coinfection in children and antiviral therapy. Expert Rev. Anti-Infect. Ther..

[26] Celik D., van Bremen K., Breitschwerdt S., Elamouri F., Swan T., Boesecke C., Rockstroh J.K., Ingiliz P. (2024). Hepatitis delta in HIV/ hepatitis B coinfection: A call for action. HIV Med..

[27] Păcurar D., Dinulescu A., Jugulete G., Păsărică A.S., Dijmărescu I. (2024). Hepatitis B in Pediatric Population: Observational Retrospective Study in Romania. Life.

[28] Yen D.W., Soriano V., Barreiro P., Sherman K.E. (2023). Triple Threat: HDV, HBV, HIV Coinfection. Clin. Liver Dis..

[29] Yano Y., Sato I., Imanishi T., Yoshida R., Matsuura T., Ueda Y., Kodama Y. (2024). Clinical Significance and Remaining Issues of Anti-HBc Antibody and HBV Core-Related Antigen. Diagnostics.

[30] Lungu G.N., Diaconescu G.I., Dumitrescu F., Docea A.O., Mitrut R., Giubelan L., Zlatian O., Mitrut P. (2024). FibroScan^®^ versus Biochemical Scores: A Study of Liver Fibrosis in HIV with HBV Co-Infection. Microorganisms.

[31] European Association for the Study of the Liver (2023). EASL Clinical Practice Guidelines on hepatitis delta virus. J. Hepatol..

[32] d’Arminio Monforte A., Tavelli A., Salpini R., Piermatteo L., D’ANna S., Carrara S., Malagnino V., Mazzotta V., Brancaccio G., Marchetti G.C. (2024). Determinants of worse liver-related outcome according to HDV infection among HBsAg positive persons living with HIV: Data from the ICONA cohort. Liver Int..

[33] European AIDS Clinical Society (2023). EACS Guidelines Version 12.0.

[34] (2025). EACS Guidelines Version 13.0. https://eacs.sanfordguide.com/en/eacs-hiv/eacs-section4/hiv-hep-co-infection/treatment-of-hbv-hiv-co-infection.

[35] de Lédinghen V., Fougerou-Leurent C., Le Pabic E., Pol S., Alfaiate D., Lacombe K., Hilleret M.-N., Lascoux-Combe C., Minello A., Billaud E. (2024). Treatment with bulevirtide in HIV-infected patients with chronic hepatitis D: ANRS HD EP01 BuleDelta and compassionate cohort. JHEP Rep..

[36] Wranke A., Hardtke S., Heidrich B., Dalekos G., Yalçin K., Tabak F., Gürel S., Çakaloğlu Y., Akarca U.S., Lammert F. (2020). Ten-year follow-up of a randomized controlled clinical trial in chronic hepatitis delta. J. Viral Hepat..

[37] Bardak A.E., Ozturk N.B., Gurakar M., Sequeira L., Yildiz E., Ozmert E.H., Idilman R., Gurakar A. (2025). Updates on Recent Advancements in Hepatitis D Virus Treatment. Viruses. Viruses.

[38] Pisaturo M., Russo A., Grimaldi P., Martini S., Coppola N. (2024). Current and future therapeutic options for chronic hepatitis D virus infection. Front. Cell. Infect. Microbiol..

